# Cytotoxic and Inflammatory Effects of Electronic and Traditional Cigarettes on Oral Gingival Cells Using a Novel Automated Smoking Instrument: An In Vitro Study

**DOI:** 10.3390/toxics10040179

**Published:** 2022-04-06

**Authors:** Liza L. Ramenzoni, Andreas Schneider, Stephan C. Fox, Michael Meyer, Mirko Meboldt, Thomas Attin, Patrick R. Schmidlin

**Affiliations:** 1 Clinic of Conservative and Preventive Dentistry, Center of Dental Medicine, University of Zurich, 8032 Zurich, Switzerland; mr.m.meyer@gmail.com (M.M.); thomas.Attin@zzm.uzh.ch (T.A.); patrick.Schmidlin@zzm.uzh.ch (P.R.S.); 2 Product Development Group Zurich, Material and Fabrication, Department of Mechanical and Process Engineering, Swiss Federal Institute of Technology Zurich, 8032 Zurich, Switzerland; anschnei@student.ethz.ch (A.S.); sfox@ethz.ch (S.C.F.); meboldtm@ethz.ch (M.M.)

**Keywords:** human epithelial gingival cells, electronic cigarette, in vitro toxicity, puff topography

## Abstract

Information about the potential oral health effects of vaping from electronic cigarettes (e-cigs) is still sparse and inconsistent. The purpose of this study was to compare the safety and cytotoxicity of e-cig liquid aerosols versus traditional cigarette (t-cig) smoke on human epithelial oral cells. T-cig smoke and e-cig aerosols were generated by a newly developed automated smoking instrument in order to simulate realistic user puffing behaviors. Air–liquid interface transwell cell cultures were exposed to standardized puff topography (puff duration: 2 s, puff volume: 35 mL, puff frequency: 1 puff every 60 s) of reference t-cigs or commercially available e-cigs at different air dilutions. Cell viability, morphology, and death rate were evaluated with MTT and TUNEL assays. The inflammatory cytokine gene expression of inflammatory genes was assessed by quantitative RT-PCR. E-cigs and t-cigs indicated similar adverse effects by enhancing cytotoxicity and cell death in a dose-dependent manner. E-cig aerosol and t-cig smoke treatment expressed upregulation of inflammatory cytokines up to 3.0-fold (*p* < 0.05). These results indicate that e-cig smoking may contribute to oral tissue–cell damage and tissue inflammation. Our approach allows the production of e-cig aerosol and t-cig smoke in order to identify harmful effects in oral tissues in vitro.

## 1. Introduction

Electronic cigarettes (e-cigs) or battery-operated cigarette products have showed an exponential increase in consumption during the last decade and have been considered an epidemic among young adults [1]. E-cigs deliver an aerosol suspension of fine liquid droplets when vaping, as well as solid particles within the vapor/gas which mainly consists of nicotine, flavors, and other chemicals, such as variable levels of aldehydes, carbonyls, propylene glycol, and vegetable glycerin [2]. Information about the harmful oral health effects of vaping is currently scarce [3]. In addition, e-cigs are commonly marketed as a safer alternative to traditional cigarettes (t-cigs), as well as a smoking substitution or cessation tool [2,3,4]. Despite its purported efficacy as a harm-reduction strategy for quitting tobacco smoking, e-cigs remain a contentious public health problem. Solid research proving that e-cigs are “less harmful” is still unavailable, therefore, dual usage of e-cigs and tobacco cigarettes is still common [4]. There is no evidence that e-cigs help smokers quit [1]. In fact, a growing body of evidence suggests that electronic cigarettes can serve as a gateway to tobacco product usage in both adults and children [5,6].

More knowledge is needed to better understand the exposure implications of e-cig aerosols and their potential for harmful oral consequences. Although e-cig aerosols contain fewer hazardous compounds than traditional cigarettes, they still contain several chemical substances which are not harmless [7,8]. E-cig aerosols contain propylene glycol and glycerin, the two major ingredients of e-liquids, as well as volatile organic compounds, carbonyls, and metals [9]. The features of aerosols are determined by the changeable elements of the e-liquids, such as flavors and nicotine content [10]. Aerosol composition is influenced by the temperature of the heating coil, the physical properties of the formulation (viscosity, wettability, etc.), and its specific heat capacity [10]. To determine the harmful e-aerosol effects on oral tissues, realistic vaping studies with representative puff topography are still required. Several studies have analyzed the effects of e-cigs on lung cells with the goal to evaluate the toxic effects on different cells and tissues [11,12,13]. A number of negative outcomes on different tissue culture systems (cell death, impaired repair, oxidative stress) have already been reported [11,12,13,14]. However, it is unclear whether the previously observed correlations are applicable to oral or tissues cell lines. In parallel, several studies compared e-cigs with t-cigs and often found increased acute toxicity [14,15,16,17,18,19,20]. E-cig aerosols prompt DNA strand breaks in vitro [21] and are known to induce oxidative modifications of DNA by free radicals [22]. A recent study showed differences in gene expression in differentiated bronchial epithelial cells between t-cig- and e-cig-exposed cells, with and without nicotine [23]. The study also showed differences in gene expression signatures in various pathways like phospholipid and fatty acid triacylglycerol, which were significantly enriched after e-cig exposure. Screening of an array of cytokines released from the cells exposed to electronic cigarette vapors or aerosols without additives showed that the basal components alone could also induce the release of several cytokines and pro-inflammatory mediators, suggesting the even humectants might have potential harmful effects [22,23]. The specific impact of these cytokines on oral tissues has yet to be defined since much past research has focused on characterizing the harmful effects of inhaled vapors on the human airway epithelium. There is also no way of confirming how similar the negative effects of e-cig aerosols are to those of t-cig smoke.

In the present study, the acute effects of e-cig aerosols and t-cig smoke on toxicity, death, and inflammation response of human oral epithelial gingival cells were evaluated. For this purpose, we specially developed a novel automated smoking machine to perform the realistic envisaged in vitro experiments in a standardized manner.

## 2. Materials and Methods

### 2.1. Air–Liquid Interface Cell Culture

Immortalized HGEK-16 cells were donated by the Oral Microbiology Institute, Clinic of Conservative and Preventive Dentistry, Center of Dental Medicine, University of Zurich, Zurich, Switzerland [24]. The cells were seeded on transwell 24-well plates (Corning Inc., Kennebunk, ME, USA) at a density of 2–2.5 × 10^5^ cells/well. All experiments were performed on transwell 24-well plates and inserts with pore size of 0.4 μm [25,26]. The cells were cultured in an incubator (5% CO_2_, 95% air at 37 °C) with keratinocyte growth medium (Provitro, Berlin, Germany) containing fibroblast grow factor, epidermal growth factor, Ca^2+^ < 0.1 mM, and insulin, without bovine pituitary extract and hydrocortisone (Merck KGaA, Darmstadt, Germany). After reaching confluence, the medium was changed to serum-free growth medium in the lower compartment and removed in the upper compartment. Cell passage was performed at regular intervals depending on cell growth characteristics using 0.25% trypsin (Seromond Biochrom, Berlin, Germany). The cell culture inserts were tested in triplicates and three independent experiments were performed for each analysis. Only cells between passages 10 and 15 were used.

### 2.2. Automated Smoking Machine

**Figure 1:**
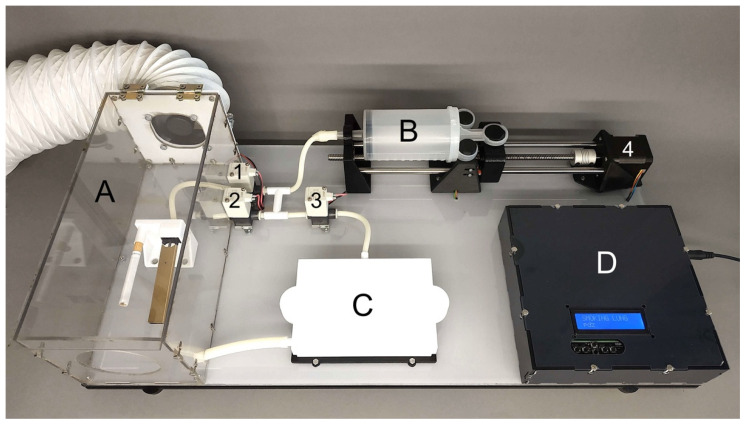
Smoking Lung device. (**A**) Smoking chamber with the cigarette holder. (**B**) Syringe pump for puff creation and dilution. (**C**) “Lung” = exposure chamber for 24-well plates. (**D**) Box for microcontroller and LCD screen for parameter selection. (**1**) Smoke valve. (**2**) Air and exhaust valve. (**3**) Exposure valve and (**4**) motor.

**Figure 2:**
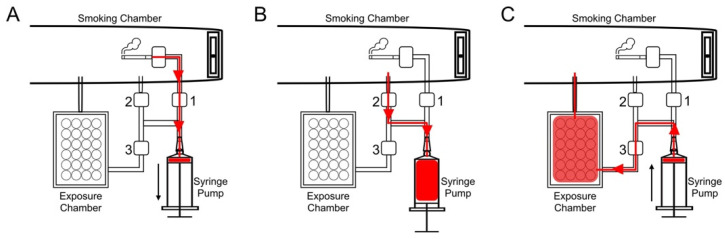
Schematic process of the dilution. (**A**) Open smoke valve (1) to generate puff. (**B**) Open air valve (2) to draw in air to dilute the smoke. For higher dilutions, part of the smoke—air mixture was pushed out of the air valve (2) and additional air was drawn in via the air valve (2). (**C**) Open exposure valve (3) to direct the smoke–air mixture into the exposure chamber and onto the cells. At the end of the cycle, an additional push with fresh air was used to remove the smoke out into the smoking chamber.

In order to create the most critical functionalities of a smoking machine and still enable reliable and comparable in vitro smoke/aerosol cell exposures, a custom-made smoking machine was developed by the Product Development Group Zurich from the Swiss Federal Institute of Technology. In the proposed setup, t-cig smoke or e-cig aerosols are generated in chamber A and drawn to the dilution chamber B with the help of a motor (4) (Figure 1). The smoke/aerosol is diluted to a desired ratio with particle-free air in chamber B, and it is blown to the cell samples in chamber C. A 3D-printed syringe pump generates the smoke and a serial dilution system dilutes the smoke or aerosol with air. Then, the smoke is led to a 3D-printed exposure chamber and directly onto the cells. The excess smoke is collected and safely discarded. The dilution system is based on serial dilution with a syringe pump (Figure 2) with three valves controlling the flow direction. The syringe pump is connected to the cigarette holder via a smoke valve (1). The puff is generated by opening the smoke valve and drawing in smoke in the volume defined by the puff regimen. Then, an air valve (2) is opened and fresh air is drawn in to generate the dilution. For higher dilutions, some of the smoke/aerosol–air mixture is pushed out via the air valve, which also serves as an exhaust valve, and additional fresh air is drawn in again and repeated until the final dilution is achieved. At the end, the diluted smoke/aerosol–air mixture is directed through an exposure valve (3) to an exposure chamber and onto the cells (Figure 2).

### 2.3. Cigarette Smoke and Aerosol Cell Exposure on the Automated Generation System

Air–liquid interface HEGK cell cultures were exposed to t-cig smoke and e-cig aerosol forms by using a puff topography profile following the ISO 3308:2015 protocol for testing in vitro cell response, as previously described [25,26,27]. The ISO 3308:2015 protocol represents a puff topography derived from human vaping behavior (puff duration: 2 s, puff volume: 35 mL, puff frequency: one puff every 60 s). In order to assess the in vitro cell toxicity, cell cultures were exposed to the smoke or aerosols with the help of the automated smoking machine described above. First, the HEGK cell cultures were exposed in triplicate to whole smoke generated from a t-cig (1R6F, Kentucky research reference cigarettes, Center of Tobacco Reference Products, University of Kentucky, Kentucky, USA) and drawn into the transwell 24-well plates chamber. The lids were removed from tissue culture dishes and serum-starved HEGK cells were placed in the smoking machine along with the smoke from one reference t-cig. The t-cig smoke was diluted with fresh air to different dilutions (1:2, 1:10, 1:50, 1:100); the exposure duration was 6.5 min per cigarette with 1 h rest between each cigarette. Then, in a new independent experiment, freshly grown triplicates of HEGK cell cultures were exposed to aerosol generated from a commercially available e-cig (JUUL C1 device, Juul Labs, Inc., San Francisco, CA, USA) and drawn into the transwell 24-well plates chamber. The dilutions (1:2, 1:10, 1:50, 1:100) and exposure duration (6.5 min per cigarette with 1 h rest) used for the t-cig were similarly employed for the e-cig exposure. The different fresh air dilutions (1:2, 1:10, 1:50, 1:100) were chosen since the puff volume of 35 mL from the ISO 3308:2015 puff topography is approximately 1/100 of a normal human lung [25,26,27]. The E-cig JUUL C1 device model (vaporizer battery-powered e-cig, pod-closed system vape category) available on the Swiss market was selected based on its popularity. Each cartridge comprises propylene glycol, glycerin, flavorings, and nicotine salts in its chemical composition (protonated nicotine). The flavor of the JUUL cigarette used in our study was “glacier mint”. The operating temperature limit of 100 °C was chosen for the e-cig (typically chosen from a range of 100 °C to 315 °C). Following the JUUL producer report, each 5.0% (nicotine-by-weight) JUUL pod or cartridge contained 0.7 mL with 5.0% nicotine-by-weight and approximately 40 mg nicotine per pod. Regarding the 1R6F t-cig, the certificate of analysis (University of Kentucky, Center for Tobacco Reference Products, Lexington, Kentucky) confirmed that the value of nicotine was 0.7 mg/cigarette, which was obtained by using the ISO 3308:2015 puff topography protocol. The 1R6F research t-cigs were stored at 4 °C and left to equilibrate at room temperature before use. The triplicate cell culture inserts were exposed in three independent exposure runs with the 1R6F t-cig or the JUUL C1 e-cig. The smoke and/or aerosol produced were blown into a dilution chamber of 200 mL volume and immediately diluted with particle-free air. The dilution air was delivered directly to the dilution chamber. An outlet was used to blow out any extra smoke or aerosol. In parallel with each smoke/aerosol exposure, fresh triplicate cell culture inserts were exposed to clean fresh air under identical circumstances and used as a negative control. The triplicate cell inserts were left untreated as an additional negative control to be used as baseline comparison for endpoint studies.

### 2.4. Cell Viability Assay

For cell viability (cytotoxicity), the cells were seeded at a concentration of 2.5 × 10^5^ cells/well on transwell 24-well plates. Then, the cells were exposed to smoke from one t-cig or e-cig using the ISO 3308:2015 puff topography protocol. The t-cig smoke or e-cig aerosol was diluted to maximum 1:100 and was added directly to the cell culture air–liquid interface. The viability was determined through a colorimetric MTT staining assay according to the manufacturer’s protocol (MTT: 3-[4,5-dimethylthiazol-2-yl]-2,5-diphenyltetrazolium bromide; Sigma-Aldrich, Steinheim, Germany), as previously described [28]. The MTT measures the activity of nicotinamide adenine dinucleotide phosphate (NADPH)-dependent cellular oxidoreductase enzymes to reduce the tetrazolium dye MTT to its insoluble formazan, which is purple in color. The viability measured by the MTT assay was read with an absorbance microplate reader (Molecular Devices, Sunnyvale, CA, USA) at a test wavelength of 595 nm with a reference wavelength of 690 nm. The optical density was calculated as the difference between the reference wavelength and the test wavelength. In total, the three independent experiments were conducted in triplicates.

### 2.5. Morphology and Cell Death Analysis

Cell morphology was evaluated by light microscopy at 0 h and 24 h after exposure to e-cig aerosol or t-cig smoke. Cell death was accessed with TUNEL assays by using the kit manufacturer’s protocol (Roche, Indianapolis, IN, USA). HEGK cells exposed to smoke/aerosol from e-cig or t-cig (1:2 dilution) were fixed in acetic acid at −20 °C. The fixed cells were incubated with TUNEL reaction mixture (terminal deoxynucleotidyl transferase and nucleotide mixture) for 1 h at 37 °C, followed by the addition of peroxidase-conjugated detection antibody. The DNA fragments were stained with 3,30-diaminobenzidine (DAB) as the substrate for the peroxidase. The DAPI stain method was performed by fixing the cells in 4% paraformaldehyde, followed by incubation in 1 g/mL DAPI solution for 30 min in the dark. The three experiments were done separately. The cells were then examined under a fluorescence microscope (Zeiss, Oberkochen, Germany).

### 2.6. Inflammatory Gene Expression Analysis

The released inflammatory mediators were measured after smoke or aerosol exposure onto the air–liquid interface HEGK cell cultures (in 1 mL per well medium stored at −80 °C). Gene expression of interleukin *(IL)-1α, IL-1β,* and *IL-6* was measured by quantitative real-time PCR (qRT-PCR). Briefly, the total ribonucleic acid (40 ng) was isolated using TRIzol reagent, RNeasy Mini kit (QIAGEN, Hilden, Germany) and quantified using NanoDrop ND-1000 (Thermo-Fisher Scientific, Wohlen, Switzerland) after smoke/aerosol exposure in four different dilutions (1:2, 1:10, 1:50, 1:100). Next, cDNA was synthesized using an iScript kit (Bio-Rad, Hercules, CA, USA). The qRT-PCR reactions were carried out on a CFX96 real-time PCR system (Bio-Rad, Hercules, CA, USA) by initial incubations of 2 min at 50 °C and 10 min at 95 °C, followed by 40 cycles of 15 s at 95 °C and 1 min at 60 °C and run in a total reaction volume of 15 μL, containing 7.5 μL of TaqMan’s one-step Master Mix kit (Applied Biosystems), 6 μL of sample (1 ng), and 1.5 μL of primer solution of 1μM (mixture of forward and reverse primers). Primer sequences and specificity for genes encoding *IL-1α, IL-1β* and *IL-6* were designed from Primer3 (version 0.4.0) and online NCBI/Primer-BLAST tool (http://www.ncbi.nlm.nih.gov/tools/primer-blast (accessed on 8 May 2019) (Table 1). Each sample contained pooled messenger ribonucleic acid from TRIzol extractions collected from the cell cultures exposed with and without smoke/aerosol at different dilutions. All samples were tested in triplicates and three independent experiments were performed. The 2^−∆∆ct^ method was used to calculate gene expression levels relative to glyceraldehyde 3-phosphate dehydrogenase (*GAPDH*) and normalized to the negative control cells.

### 2.7. Statistical Analysis

Comparisons between groups were conducted by multiple comparison analysis of variance (ANOVA) with Bonferroni adjustment with a global significance level of 5% using the SPSS software (IBM SPSS Statistics for Windows, version 23.0; IBM Corp., Armonk, NY, USA). The mean values and standard deviations were computed for the MTT test. Results were expressed as means standard error of the mean. The differences were considered statistically significant at *p* < 0.05. All experiments were performed in triplicate and repeated at least three times under the same conditions.

## 3. Results

### 3.1. Cell Viability

**Figure 3:**
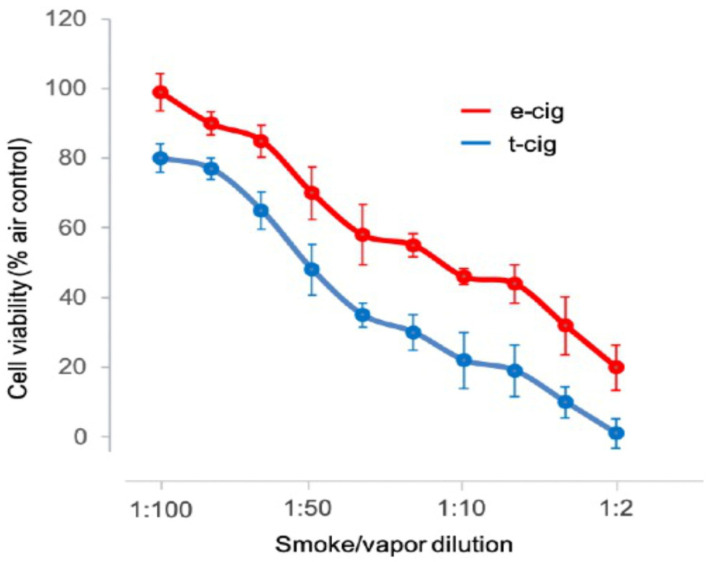
Cell toxicity presented by cellular viability assay (MTT). Human epithelial gingival keratinocytes were exposed to several increased dilutions of aerosol and smoke from an e-cig and t-cig compared to fresh air (negative control). *Y*-axis = OD (optical density), *x*-axis = dilutions.

Quantitative MTT analysis detected a decrease in cell viability and appearance of dead cells immediately after 6.5 min exposure to one puff topography (ISO 3308:2015 protocol: puff duration: 2 s, puff volume: 35 mL, puff frequency: one puff every 60 s) of the e-cig aerosol compared to the t-cig smoke. There was a statistically significant (*p* < 0.05) increase in cell toxicity upon exposure to the e-cig device aerosols compared to the t-cig smoke and compared to the untreated air controls at 1:2, 1:10, 1:50, 1:100 (Figure 3). The HEGK cells were not affected by exposure to fresh air (negative control) and MTT values were similar to those of untreated cells. The results showed the adverse toxic effects of e-cig aerosols on normal cells with observed apoptotic morphology. The results obtained indicated that a single exposure to the e-cig aerosol increased cell death and was well correlated with increasing concentration of the aerosol (Figure 3).

### 3.2. Cell Death

**Figure 4:**
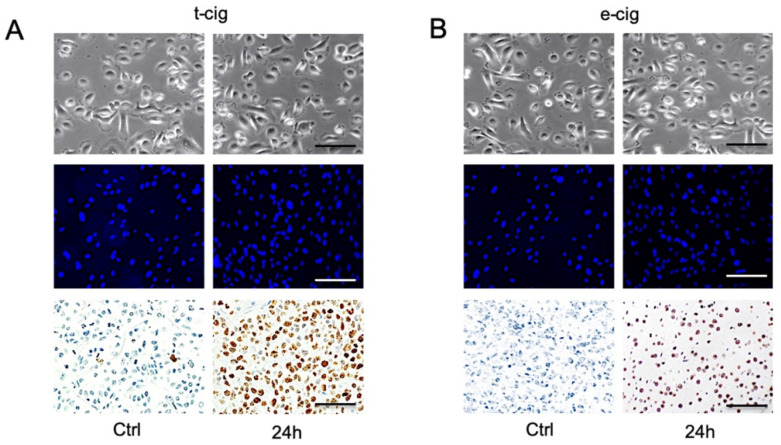
Apoptotic cells and their morphology measured by TUNEL assay and DAPI staining. (**A**) t-cig smoke and (**B**) aerosol e-cig. Phase-contrast microscopy (**upper**), DAPI staining nuclei (**middle**), and TUNEL-positive cells stained as dark brown (**lower**). Scale bar = 100 μm.

When compared to the untreated negative controls, apoptotic morphology was detected after 24 h of exposure to t-cig smoke and e-cig aerosol, including apoptotic cellular bodies, nuclear condensation, DNA fragmentation, and perinuclear apoptotic features (Figure 4). The untreated cells did not show apoptotic features. Both smoke (t-cig) and aerosol (e-cig) exposure increased the TUNEL positive cells (stained in dark brown) 24 h after treatment compared to the negative control cultures without exposure (Ctrl). Additionally, apoptotic cellular bodies under phase-contrast microscopy and DAPI staining revealed induced condensation of nuclei (Figure 4). However, no significant difference was found for cell death or changes in cell morphology between t-cigs and e-cigs.

### 3.3. Gene Expression

**Figure 5:**
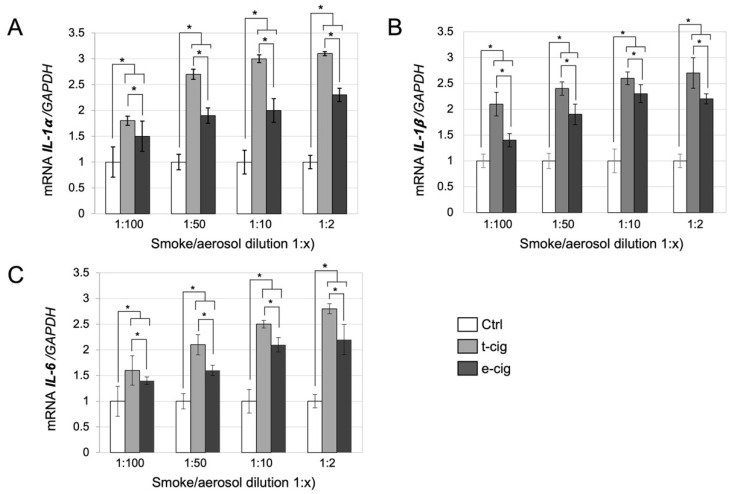
Inflammatory cytokine gene expression by HEGK exposed to the negative control and four increased dilutions of aerosol/smoke from the e-cig and t-cig (1:2, 1:10, 1:50, 1:100). (**A**) *IL-1α*, (**B**) *IL-1β*, (**C**) *IL-6.* Means ± SD, * *p* < 0.05.

The RT-qPCR results showed that both e-cig aerosol and t-cig smoke treatments comparatively increased the expression of *IL-1α, IL-1β,* and *IL-6* in all used aerosol/smoke dilutions (1:2, 1:10, 1:50, 1:100) compared with the untreated negative control cells (Figure 5). The t-cig smoke treatment induced the highest and most significant levels of all pro-inflammatory cytokine expressions tested in this study (upregulation up to 3 folds, *p* < 0.05) compared to e-cig aerosol treatment (upregulation up to 2.5 folds, *p* < 0.05). Nevertheless, e-cigs also presented high levels of inflammatory cytokines compared to the untreated negative controls (*p* < 0.05). Lower dilutions of t-cig smoke and e-cig aerosol (1:10 and 1:2) prompted significantly higher levels of *IL-1α, IL-1β*, and *IL-6* compared to the untreated control (*p* < 0.05). The observed effect on inflammatory expression was well correlated with increasing concentration of t-cig smoke and e-cig aerosol.

## 4. Discussion

In the present study, we established an interdisciplinary approach, where we could combine experts in product development and cell biology, in order to create a simple but reproducible procedure to assay the effects of different consumption methods of vaporized nicotine on cell systems. The study’s key finding is that e-cigs have an effect on oral epithelial cells by enhancing cell toxicity and inflammatory mediator release. Nonetheless, compared to t-cig smoke exposure, the acute toxic effect of e-cig aerosol appears to be smaller. According to cytokine expression analysis, the expression patterns of t-cig-exposed cells were higher than those of e-cig-exposed cells.

The findings from the t-cig-exposed cells are consistent with prior findings that t-cig smoking causes a deficiency in host defenses and epithelial barrier breakdown in vivo and in vitro [29,30,31,32,33]. It has been suggested that vaping e-cigs stimulates cytokine release and may elicit cell toxicity and inflammation in the nasal epithelial mucosa [34]. In fact, e-cig usage has been linked to reduction in the expression of immune-related genes [34,35,36]. The administration of e-cig fluid, rather than vapor, to primary epithelial cells has been demonstrated to increase inflammation and virus infection susceptibility [37]. Furthermore, inhaling smoke or other noxious substances was frequently linked to inflammation, which was linked to host resistance, systemic reaction, and repair [37]. There is substantial amount of evidence that t-cig exposure induces airway epithelial cells to produce a combined assortment of inflammatory mediators [36,37,38,39], but the molecule responsible has yet to be identified. T-cig smoke and e-cig aerosol boosted the synthesis and expression of pro-inflammatory mediators in the current investigation. Our findings are consistent with previous research on human airway epithelial cells [36,37,38,39]. We investigated the gene expression of the differentiated gingival epithelium following t-cig and e-cig exposure to acquire a thorough perspective of the differentially regulated inflammatory genes. When compared to t-cig-exposed cells, the changes in gene expression of the e-cig-exposed cells were fewer, but they were always higher than the controls. Another notable pro-inflammatory mediator—IL-1, was detected among the genes with the largest variation in gene expression and the highest upregulated genes in the t-cig group. E-cigs are known to alter the metabolome of epithelial cells, causing major changes that partially overlap with t-cigarette effects [40].

Smoking is considered one of the main causes of various oral diseases [41]. According to studies, smokers have an increased risk of acquiring oral cancer (i.e., oral squamous cell carcinoma), xerostomia, nicotinic stomatitis, melanosis, hairy tongue, leukoplakia, and oral candidiasis [41,42]. In addition, many smoking-related complications consist of a large range of periodontal problems, tooth lost, teeth decay, and wound healing impairment [43]. In fact, compared to non-smokers, smoking tobacco or vaping has increased the prevalence and severity of periodontal disease, as well as the reduction rate of implant survival [43]. The periodontal ligament and oral gingival epithelium are crucial abundant cells in the periodontal tissue, which are direct targets of vaping and e-cig use. It is known that upon stimulatory stress, these cells are particularly able to produce and regulate inflammatory response. However, most of the studies mainly focus on the toxic effects of e-cigs on the respiratory airway epithelium. Few studies have established the harmful effects of e-cigarette vaping, particularly in vitro, in response to inflammatory oral gingival epithelial cells. Thus, our observed results depict a valuable representation on how the use of new forms of tobacco products, such as e-cig, may have an intrinsic link to oral periodontal diseases. Other previous data have shown that e-cigs can induce inflammatory cytokines, wound healing arrest, and influence oxidative stress similar to t-cigs [44,45]. Similar to our in vitro findings, other authors have demonstrated corresponding harmful effects of e-cigs on gingival fibroblasts and periodontal ligament cells [44,45,46]. These outcomes in critical cells of the oral cavity highlight the importance of increasing our knowledge of the effects of e-cig aerosols. Nevertheless, a major knowledge gap still exists, including effects of nicotine, tobacco smoke, or aerosol in systemic versus local effects of e-cigs in the oral cavity, with variation on levels of other chemicals and flavorings on oral wound healing and disease. To address these gaps, the approach outlined in this study should be undertaken to compare t-cig and e-cig effects on histologic and immunohistochemical changes in bones and soft tissues, as well as molecular effects after tobacco or vapor exposure. The future development of different in vitro oral cell analysis should always attempt to simulate oral smoke inhalation to ensure clinical relevance, which may have a public education impact on e-cig product consumption.

The present and other studies have—as always—limitations. To begin with, in vitro investigations only provide data on short-term results and do not enable for in vivo long-term effects of e-cigarette usage to be predicted. As a result, no conclusions can be drawn about the long-term safety of e-cigs or the potential for harm reduction. In addition, this study only focused on a specific cell line, i.e., oral gingival keratinocytes, while other studies focused on other specific cellular systems. Notably, our group put the main focus on an oral surrogate cell model. The availability of flavors as additives for e-cig vapor and various forms of vaporizing devices is rising and it is unregulated; flavors were not addressed in the current study. Flavors, particularly cinnamon-containing beverages, have been proven to cause toxicity in vitro and in animals [12]. Comparing two physically dissimilar agents as e-cig aerosol and t-cig smoke and attempting to normalize is, however, a difficult task. Nicotine is one of the few chemicals in both traditional and electronic cigarettes that is associated to toxicity or addiction. Additionally, standardizing on nicotine consumption seems sensible because the user will require specific amounts of nicotine, making the total uptake of t-cigs and e-cigs comparable [47].

## 5. Conclusions

To summarize, the current study tested for the first time an innovative smoke/aerosol generation system specifically developed for e-cigs and t-cigs, which allowed the generation of representative vapor produced under real simulation conditions from e-cig devices, making it particularly well suited for realistic inhalation toxicology studies in vitro. We were able to confirm the adverse toxic effects of e-cig aerosols on human epithelial gingival cells leading to apoptotic morphology. In addition, an increase in toxicity and upregulation of inflammatory interleukin genes were detected. The results indicate that even a short-term single exposure to e-cig aerosols may affect epithelial morphology resulting in increased cell death. While the overall impact of e-cigs on epithelial cells appears to be less hazardous, in general, than those of t-cigs, in vitro data do not allow for judgments on long-term safety. On the other hand, e-cigs should not be regarded as “harmless” at this time.

## Figures and Tables

**Table 1:** Primer sequences and properties.

Gene	Orientation	Sequence (5′→3′)	Tm (°C)	Annealing Temperature (°C)	Product Size (bp)
*IL-1α*	Forward	TGCCTATGTCTCAGCCTCTT	58.13	64.3	642
	Reverse	GAGGCCATTTGGGAACTTCT	57.78		
*IL-β1*	Forward	TAGAGCTGCTGGCCTTGTTA	58.72	64.8	210
	Reverse	ACCTGTAAAGGCTTCTCGGA	58.36		
*IL-6*	Forward	ATGAACTCCTTCTCCACAAGC	57.94	64.1	264
	Reverse	GTTTTCTGCCAGTGCCTCTTTG	60.54		
*GAPDH*	Forward	GCTCTCTGCTCCTCCCTGTT	61.26	65.9	374
	Reverse	CACACCGACCTTCACCATCT	59.68		

Tm, melting temperature.

## Data Availability

The data presented in this study are available on request from the corresponding author. The data are not publicly available due to institutional and national datasharing restrictions.
